# Regulation of cargo selection in exosome biogenesis and its biomedical applications in cancer

**DOI:** 10.1038/s12276-024-01209-y

**Published:** 2024-04-05

**Authors:** Yu Jin Lee, Kyeong Jin Shin, Young Chan Chae

**Affiliations:** 1 Department of Biological Sciences, Ulsan National Institute of Science and Technology (UNIST), Ulsan, 44919 Republic of Korea; 2 National Creative Research Center for Cell Plasticity, KAIST Stem Cell Center, Department of Biological Sciences, KAIST, Daejeon, 34141 Republic of Korea

**Keywords:** Cell signalling, Cancer microenvironment

## Abstract

Extracellular vesicles (EVs), including exosomes, are increasingly recognized as potent mediators of intercellular communication due to their capacity to transport a diverse array of bioactive molecules. They assume vital roles in a wide range of physiological and pathological processes and hold significant promise as emerging disease biomarkers, therapeutic agents, and carriers for drug delivery. Exosomes encompass specific groups of membrane proteins, lipids, nucleic acids, cytosolic proteins, and other signaling molecules within their interior. These cargo molecules dictate targeting specificity and functional roles upon reaching recipient cells. Despite our growing understanding of the significance of exosomes in diverse biological processes, the molecular mechanisms governing the selective sorting and packaging of cargo within exosomes have not been fully elucidated. In this review, we summarize current insights into the molecular mechanisms that regulate the sorting of various molecules into exosomes, the resulting biological functions, and potential clinical applications, with a particular emphasis on their relevance in cancer and other diseases. A comprehensive understanding of the loading processes and mechanisms involved in exosome cargo sorting is essential for uncovering the physiological and pathological roles of exosomes, identifying therapeutic targets, and advancing the clinical development of exosome-based therapeutics.

## Exosome cargo sorting: insights into cellular communication and disease

Extracellular vesicles (EVs)—tiny structures produced by cells that are important for cell-to-cell communication—contain various molecules, a process known as cargo sorting. However, how these molecules are selected and packaged into EVs is not fully understood. This research by Lee, Shin, and Chae provides a detailed analysis of our current knowledge of cargo sorting in exosome biogenesis. They reviewed the literature on the types of molecules in exosome cargo, the factors and machinery controlling cargo selection and packaging, and the role of cargo sorting in diseases. They found that understanding exosome cargo sorting is vital for managing diseases and developing exosome-based treatments. This could help design exosomes that can more effectively deliver therapeutic substances to specific cells or tissues, improving the effectiveness and accuracy of these treatments in clinical use.

This summary was initially drafted using artificial intelligence, then revised and fact-checked by the author.

## Introduction

### Overview of extracellular vesicles (EVs)

EVs represent a diverse class of cell-derived membranous structures that play pivotal roles in regulating intercellular communication. These vesicles are involved in transmitting bioactive molecules and modulating various physiological and pathological processes. There are several subtypes of EVs, including exosomes, which were first characterized through electron microscopy. Initially, these EVs were considered waste bags responsible for expelling unwanted cellular products outside of the cell. Notably, transferrin was the first cargo discovered within these EVs^1^.

However, beginning in the early 2000s, the advent of advanced analytical methods, such as mass spectrometry and next-generation sequencing, revealed that EVs contain a diverse array of biomolecules, including proteins, lipids, metabolites, and nucleic acids, including various types of DNA and RNA^2,3^. This revelation reshaped our understanding of exosomes and highlighted the significance of EVs in intercellular communication beyond mere cellular waste removal. Furthermore, research on EVs has rapidly expanded as their potential for diagnostic and therapeutic applications in various diseases, including neurodegenerative disorders, cardiovascular dysfunction, metabolic diseases, and immune-related conditions, including cancer, became evident^4^.

EVs are secreted into the extracellular space and taken up by target cells, thereby modulating their phenotype. In contrast to the conventional ligand‒receptor-mediated communication system, EV-mediated signaling involves the direct transfer of diverse cargo into recipient cells, thereby enabling the direct regulation of cellular functions at multiple levels, including the genetic, signaling pathway, and functional levels. As innate vesicles, exosomes shield their cargo from enzymatic degradation and exhibit reduced immunogenicity within the circulatory system, making them ideal for transporting biomaterials^5,6^. Due to their efficacy in delivering biomaterials, EVs are highly promising candidates for a wide array of biomedical applications, including drug delivery, diagnostics, and therapeutic interventions^4^.

Exosome biogenesis is a complex, highly regulated process that involves several sequential stages, from the initial formation of early endosomes to the eventual release of fully mature exosomes into the extracellular environment. Among these stages, the selective sorting of cargo molecules into nascent exosome vesicles has emerged as a critical determinant of exosome functionality, diversity, and specificity^7^. In this comprehensive review, we aim to provide an in-depth overview of our current understanding of the cargo sorting mechanisms involved in exosome biogenesis. We explore the various classes of molecules that constitute exosome cargo, the regulatory factors and machinery that govern cargo selection and packaging, and the implications of cargo sorting in disease contexts. Understanding how exosomes manage cargo sorting is a crucial objective for disease management and a foundational step in advancing sophisticated exosome-based therapies^7,8^. This approach facilitates the design of exosomes that are better equipped to transport therapeutic payloads to specific cells or tissues, thereby enhancing the efficacy and precision of EV treatments in clinical applications.

### Classification of EVs

EVs are primarily classified into three major groups based on their size and cellular origin, although EV classification is continuously evolving^7^. Within this classification, exosomes constitute the smallest EVs, typically measuring between 30 and 150 nm in diameter^9^. They originate from the endocytic pathway, specifically from late endosomes known as multivesicular bodies (MVBs), where exosome formation begins. Subsequent fusion of MVBs with the plasma membrane releases their internal vesicles into the extracellular space, resulting in exosomes^10,11^.

Exosomes can encapsulate a wide array of bioactive molecules, including microRNAs (miRNAs), proteins, and lipids. The composition of exosomal cargo is subject to regulation by various signaling pathways and external cellular conditions. Moreover, the composition is dynamic and capable of changing in response to different stimuli and cellular signals. Therefore, exosomes are considered one of the most important forms of EVs in intercellular signaling; exosomes play pivotal roles in transmitting bioactive molecules and responding to the everchanging cellular environment.

In addition to exosomes, EVs include microvesicles (MVs) and apoptotic bodies. MVs, which are larger than exosomes and range from 100–1000 nm in diameter, result from outward budding and fission of the cell’s plasma membrane. Their cargo predominantly includes cytosolic contents such as proteins, nucleic acids, lipids, and metabolites. However, the absence of specific markers for distinguishing MVs from exosomes remains a challenge^3,12^.

Apoptotic bodies, which make up the largest of the three EV groups, typically measure between 1,000 and 5000 nm. They emerge during apoptosis when cells fragment into smaller units and enveloped by membrane-bound vesicles. These vesicles contain DNA fragments, histones, chromatin remnants, cytosolic fractions, and degraded proteins. Typically, apoptotic bodies are cleared by phagocytic cells^7,12^.

Other types of EVs with potentially specialized functions have also been described. Large oncosomes (1 μm) and ARRDC1-mediated microvesicles (ARMMs) arise from outward protrusions of the plasma membrane that are excised and shed into the extracellular space. Recently, migrasomes derived from retraction fibers have been described and characterized^13^. Additionally, two nonvesicular extracellular nanoparticles (NVEPs), exomeres and supermeres, have been shown to be enriched in many cargoes previously associated with EVs. In this review, we focused on exosomes, the most studied type of EV.

### Distinct characteristics of exosomes and the composition of exosomes

**Fig. 1:**
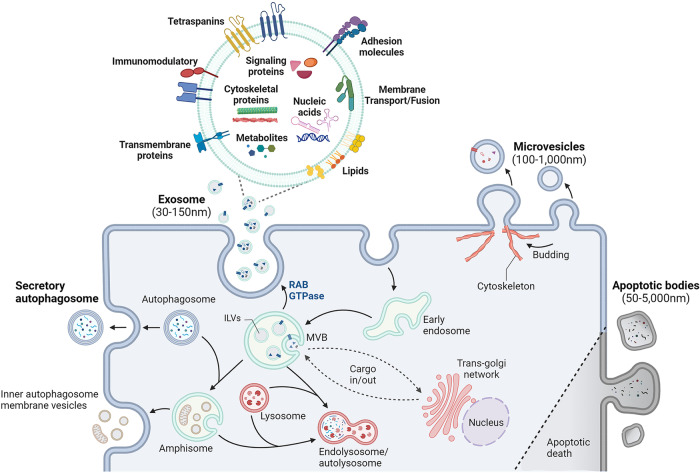
Mechanisms of extracellular vesicle (EV) biogenesis and EV components. EV biogenesis occurs through multiple pathways, including through multivesicular bodies (MVBs) within endosomes, which leads to the secretion of exosomes, and through the plasma membrane, which results in microvesicle (MV) generation. After endocytosis, early endosomes undergo maturation in MVBs, during which they form intraluminal vesicles (ILVs) through inward membrane budding. MVBs either fuse with lysosomes for degradation or dock at the cell periphery for exosome secretion, which is facilitated by the RAB GTPases and SNARE complexes. Other types of EVs include apoptotic bodies, which are released during apoptosis via membrane budding; migrasomes, which originate from retraction fibers and contain internal vesicles; and vesicles, which are formed from amphisomes and result from the fusion of outer autophagosome membranes with late-endosomes. Exosomes carry diverse macromolecules, including signaling proteins, transcriptional regulators, various RNA species, DNA, and lipids.

Exosomes have garnered significant attention due to their unique characteristics that distinguish them from other EVs (Fig. 1). The lipid bilayer membrane surrounding exosomes is composed of phosphatidylserine, phosphatidylcholine, sphingomyelin, ceramides, and cholesterol and plays a crucial role in maintaining exosome structural integrity and function^4^. Exosomes are rich in various forms of nucleic acids, including mRNAs, miRNAs, ribosomal RNAs (rRNAs), long noncoding RNAs (lncRNAs), transfer RNAs (tRNAs), small nuclear RNAs (snRNAs), small nucleolar RNAs (snoRNAs), and p-element-induced wimpy testes (piwi)-interacting RNAs (piRNAs)^14^. Exosomes can also include double-stranded or single-stranded DNA (dsDNA and ssDNA, respectively) and mitochondrial DNA (mtDNA)^15,16^.

The primary membrane-bound and cytosolic proteins incorporated in exosomes include those involved in membrane transport or fusion (Rab GTPases and annexins), proteins associated with exosome biogenesis (the ESCRT complex, ALIX, and TSG101), heat shock proteins (HSP70 and HSP90), integrins, members of the tetraspanin family (CD63, CD81, and CD82), and cytoskeletal proteins^3^. Numerous antigen and receptor proteins are expressed on the surface of exosomes. These surface proteins interact with receptors on recipient cells to initiate intracellular signaling pathways. Additionally, exosomes also feature glycoproteins and glycolipids on their surface, along with signaling molecules such as cytokines, growth factors, small molecules, and metabolites^7^.

Exosomal cargoes located on the external membrane surface can directly engage with recipient cells to initiate signaling events or facilitate specific targeting. Conversely, internal cargoes are released into recipient cells upon exosome uptake, thereby influencing cellular functions at the genetic, protein, or lipid level. The amalgamation of these external and internal cargoes renders exosomes highly versatile vehicles for intercellular communication and the transfer of biological information. Consequently, the selective sorting and packaging of specific cargo into exosomes constitute a fundamental aspect of their biogenesis and function. Understanding the intricacies of cargo sorting mechanisms in exosome formation is essential for deciphering the physiological significance of exosomes and holds great promise for advancing diagnostic and therapeutic strategies across a wide spectrum of diseases.

## Exosome biogenesis and selective cargo sorting processes

In recent years, considerable progress has been made in unraveling the mechanisms underlying the formation and secretion of exosomes. In conventional membrane trafficking pathways, cargoes destined for specific organelles typically recruit the machinery necessary for their own sorting and transport^17^. Generally, the recruited trafficking machinery isolates cargoes on patches of the membrane, reshapes the surrounding membrane into vesicular structures, and ultimately detaches vesicles from the source membrane^11^. Exosome cargoes appear to follow a comparable fundamental process.

Exosome biogenesis commences with the formation of multiple intraluminal vesicles (ILVs) within endocytic compartments known as multivesicular endosomes or MVBs^18^ (Fig. 1). ILVs emerge through the inward budding of the endosome’s limiting membrane and encapsulate cargoes destined for exosomes. These ILVs are enriched with specific cargoes and interact with trafficking effectors (Table 1) on endosomal and plasma membrane patches. These unique interactions lead to membrane bending and scission processes that give rise to exosomes. Subsequently, MVBs traverse the plasma membrane, where they fuse and release ILVs into the extracellular space as exosomes. In contrast, plasma membrane-derived MVs are generated via direct budding of the plasma membrane into the extracellular space^19^. In this section, we describe the current understanding of the basic mechanisms of exosome cargo sorting.

**Table 1:** Components that regulate cargo loading in exosome biogenesis.

Component	Cargo	Refs.
ESCRT-dependent pathways (classical)		
HRS	Ub modifications	^21^
	PD-L1	^53^
TSG101	CD63	^23^
	MHC-II	
FMRP	miR-155	^74^
ESCRT-dependent pathways (variation)		
Syntenin	CD63	^22^
	Syndecan1	
Syndecan1	β Integrin	^24,44^
	Fibronectin	
ALIX	Syntenin	^22^
	PAR1	^52^
	PD-L1	^53^
YBX1	miR-133	^72^
ESCRT-independent pathways		
CD9	CD10	^39^
	β-Catenin	
CD63	PMEL17	^38^
	LMP1	^29^
CD81	RAC	^39^
CD82	β-Catenin	^39^
	Ezrin	
Others (unknown)		
hnRNPA1	miR-27a-3p miR-27b-3p	^69^
	miR-92a-3p	
	miR-221-3p	
	miR-21	
hnRNPA2B1	miR-198	^70^
	miR-601	
	miR-451	
	miR-575	
	miR-125a-3p	
	miR-887	
	AFAP1-AS1 IncRNA	^71^
	AGAP2-AS1 IncRNA	
SYNCRIP (hnRNP-Q or NSAP1)	miR-3470a	^67^
	miR-194-2-3p	
La protein	miR-126	^14^
	miR-145	
	miR-486	
	miR-122	
	miR-142	

### ESCRT-mediated exosome biogenesis

**Fig. 2:**
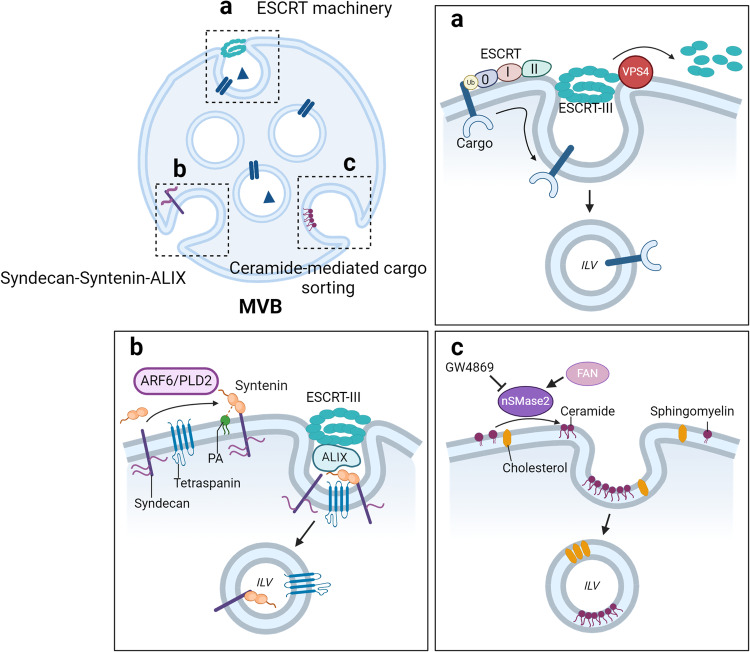
Exosome biogenesis mechanisms and cargo sorting pathways. Multiple molecular mechanisms of intraluminal vesicle (ILV) generation in multivesicular bodies (MVBs) have been revealed. **a** The classical ESCRT-dependent pathway involves the recognition of ubiquitinated proteins in the endosomal membrane by the ESCRT-0, -I, -II, and -III subcomplexes. The ATPase VPS4 cooperates in a stepwise manner to mediate ILV formation. **b** In the syndecan-syntenin-ALIX pathway, membrane budding and cargo clustering can occur independently of the early ESCRT machinery, with VPS4 required for the scission step. **c** Ceramide, which is generated from sphingomyelin by nSMase2, plays a key role in the ESCRT-independent pathway of ILV biogenesis. Ceramide can form lipid raft microdomains and trigger the conversion of ILVs into MVBs. nSMase2 is activated by FAN and can be pharmacologically inhibited by small molecules such as GW4869. The cargoes sorted through this pathway include flotillin, cholesterol, and tetraspanins, which are localized to lipid rafts.

The ESCRT pathway was one of the first pathways associated with exosome biogenesis and involves the coordinated action of all four ESCRT complexes (ESCRT-0, ESCRT-I, ESCRT-II, and ESCRT-III) in conjunction with disassembly and deubiquitylating enzymes present on the endosome membrane. This process also involves the ESCRT accessory protein VPS4^20^ (Fig. 2a).

The ESCRT-0, -I, and -II complexes are equipped with ubiquitin-binding domains that allow them to capture ubiquitinated cargo^21^, such as epidermal growth factor receptor (EGFR) and ligand‒receptor signaling complexes. Moreover, ESCRT-I, -II, and -III play crucial roles in remodeling the membrane for ILV binding. To complete exosome biogenesis, ESCRT-II induces the formation of ESCRT-III filaments, which facilitate the severing of the nascent exosome neck from the endosome membrane^17^. ESCRT-III is thought to be directed to the vesicle bud neck either by sensing negative membrane curvature or by promoting membrane bending to facilitate the separation of ILVs from the endosome membrane. However, the specific roles of ESCRT-III are still under investigation.

The AAA ATPase VPS4 interacts with ESCRT-III to facilitate the final stages of ILV formation by promoting membrane scission, resulting in the leakage of ILVs into the MVB lumens. Importantly, the deletion of multiple ESCRT protein subunits or VPS4 can significantly impact exosome biogenesis, leading to alterations in exosome number, size, and protein composition to varying extents^22,23^. The roles of ESCRT proteins in ILV biogenesis are conserved, as evidenced by studies in budding yeast, where deletion of ESCRT proteins results in prevacuolar, endosomal compartments that lack ILVs^21,24^.

### Variations in the ESCRT pathway

Exosomes carrying different cargoes exhibit distinct requirements for ESCRT proteins during biogenesis, likely reflecting differences in how cargoes are recruited into the ESCRT pathway. Defining these requirements can be challenging in mammalian cells due to the presence of multiple ESCRT protein isoforms with partially redundant functions and the pleiotropic effects of ESCRT deletion^23^. However, recent insights have shed light on such variations, as exemplified by the syndecan-syntenin-ALIX pathway^22^ (Fig. 2b).

In MCF7 breast cancer cells, syntenin, a cytoplasmic adapter protein, recruits ALIX to MVBs, where its interaction with ESCRT-III induces ILV formation^22^. Regulation of syndecan-syntenin-ALIX-mediated exosome biogenesis involves activation of the oncogenic tyrosine kinase SRC. SRC phosphorylates syndecan1, syntenin, and ALIX, thereby stimulating exosome biogenesis^25^. The recruitment of syntenin to endosomes may occur through the activation of phospholipase D 2 (PLD2)^26^ by the GTPase ADP-ribosylation factor 6 (ARF6). Subsequently, PLD2 generates phosphatidic acid at the MVB-limiting membrane, thus facilitating syntenin binding. Alternatively, phosphatidic acid generation at endosomes can result from PLD1 activation by Ral GTPases, which further promotes exosome biogenesis^27^. The localization of ALIX to MVBs is also driven by its association with the late endosome-specific lipid lysobisphosphatidic acid (LBPA), which supports ESCRT-III-dependent ILV formation and exosome production in HeLa cells^28^. Importantly, the syndecan-syntenin-ALIX pathway can be utilized by the Epstein–Barr virus to load exosomes with latent membrane protein 1 (LMP1), the major Epstein–Barr virus oncoprotein^29^.

An alternative branch of the ESCRT pathway involves the accessory ESCRT-III proteins CHMP1, CHMP5, and increased sodium tolerance protein 1 (IST1). These proteins play pivotal roles in orchestrating the creation of a distinct subgroup of exosomes within cells subjected to conditions of glutamine deprivation and/or inhibition of the mTOR-Akt pathway^30^. These exosomes originate within recycling endosomes marked by RAB11, in contract to conventional RAB5- or RAB7-positive endosomes. These investigations shed light on the role of accessory ESCRT-III proteins in the generation of stress-induced exosomes.

Furthermore, GPR143, an atypical G-protein coupled receptor, has emerged as a regulator of ESCRT-dependent exosome production. GPR143 recruits HRS (ESCRT-0) to endosomes to modulate interactions with cargo proteins. This orchestration leads to altered protein sorting in ILVs and modifications to the exosomal proteome composition. Notably, the expression of GPR143 facilitates the secretion of oncogenic exosomes that contain integrins that promote cell motility and cancer metastasis^31^.

### ESCRT-independent exosome biogenesis

Ongoing research has revealed that the formation of MVBs and ILVs is not solely contingent upon the ESCRT complex. Rather, emerging research has demonstrated the existence of ESCRT-independent mechanisms that govern exosome generation.

#### Lipids in exosome biogenesis

Exosomes are rich in cholesterol, phosphatidylcholine, phosphatidylserine, sphingomyelin, and ceramide, each of which contributes distinct functions in terms of exosome biogenesis and uptake, and these lipids have a consequential impact on recipient cells^32^. Ceramide assumes a prominent role in exosome biogenesis^33^. Recent reports indicate that certain cargo molecules can undergo ESCRT-independent sorting into ILVs through a lipid-dependent pathway (Fig. 2c). For example, proteolipid protein (PLP), an abundant membrane protein within the central nervous system^34^, exhibits a proclivity for endosomal sorting in oligodendrocytes, a propensity retained even in the absence of essential ESCRT components, such as TSG101, ALIX, or HRS. This persistence of sorting competence is observed despite interventions such as small interfering RNA (siRNA)-mediated depletion or the expression of dominant-negative VPS4^33^.

In addition to PLP, the enzyme neutral sphingomyelinase 2 (nSMase 2), which is responsible for the generation of ceramide from sphingomyelin within endosomes, serves as a promoter of ILV and exosome biogenesis. The functional involvement of ceramide within MVBs may be facilitated by diverse ancillary pathways that further amplify exosome biogenesis. The autophagy-related protein microtubule-associated protein 1 A/1B-light chain 3 (LC3) plays a role in recruiting factors associated with nSMase (FAN), an activator of nSMase, to endosome membranes. This recruitment facilitates ceramide-mediated ILV formation^35^. Furthermore, the activation of RAB31 augments both exosome production and the packaging of EGFR into exosomes derived from cancer cells^36^. Importantly, it is proposed that this augmentation occurs through the ceramide-dependent pathway of ILV production; these results underscore the potential significance of ILV in the context of cancer cell exosome biogenesis.

#### Tetraspanins in exosome biogenesis

Tetraspanins, integral membrane surface proteins frequently enriched in small EVs, prominently engage in interactions with integrins and associated proteins at the cell membrane, thus orchestrating the formation of highly organized tetraspanin-enriched microdomains (TEMs)^37^. The ‘classic’ tetraspanins that have been identified as exosomal cargoes, including CD63, CD81, and CD9, not only contribute to exosome biogenesis but also function as molecular guides for the sorting of other TEM-associated proteins into exosomes (Table 1). For example, the melanocyte-specific glycoprotein PMEL readily undergoes sorting into ILVs upon interacting with CD63^38^. Similarly, the membrane metalloproteinase CD10 engages with CD9 to facilitate its entry into ILVs. Additionally, CD9 and CD82 interact with E-cadherin to promote exosomal secretion of β-catenin. Nevertheless, efforts aimed at precisely elucidating the intricate mechanisms by which tetraspanins contribute to exosome biogenesis have yielded conflicting results.

Depending on the specific tetraspanin under consideration, its impact on exosome biogenesis may manifest redundantly; thus, specific tetraspanins may have minimal consequences for this process^39^. Alternatively, disrupting TEMs through the depletion of one tetraspanin may yield altered localization and/or protein‒protein interactions among other tetraspanins, ultimately leading to an increase in exosome biogenesis. Thus, inhibition of exosome biogenesis at another membrane site, such as the endosome membrane, may consequently promote exosome biogenesis at one membrane location, such as the plasma membrane. This phenomenon collectively culminates in an overall increase in exosome abundance^40^. Consequently, caution is needed when assessing the classification of exosomes solely based on the presence or absence of any given tetraspanin within a heterogeneous mixture of small EVs.

## Cargo sorting in exosome biogenesis

### Protein composition and enrichment in exosomes

Exosomes serve as versatile carriers for a diverse array of biomolecules, including transmembrane proteins, membrane-associated proteins, and soluble proteins sequestered within their lumens. Proteomic analyses have revealed the rich and intricate protein content of exosomes, thus highlighting their potential roles in intercellular communication and disease diagnosis.

Proteomic characterizations of EVs derived from cellular sources, including cancer cell lines, have revealed distinct protein profiles, often indicative of the cellular origins of these vesicles^41,42^. In a separate study, 497 EV preparations sourced from cell lines, tissue explants, and both murine and human plasma were evaluated, revealing commonalities in the protein signatures of these EVs^43^. These investigations have led to the identification of common proteins, some of which are associated with exosome biogenesis and tend to serve as consistent exosome markers. Furthermore, a subset of the proteome demonstrates cell-type specificity, rendering them promising candidates for disease diagnosis and patient prognosis.

Notably, the mechanisms responsible for sorting and packaging proteins into exosomes remain to be fully elucidated. However, a significant portion of these proteins are cell-surface receptors, suggesting that they may originate from processes such as plasma membrane budding or shedding. Additionally, proteomic analyses have played a pivotal role in characterizing disease stages across various cancer types, thus underscoring the potential of selective exosomal protein packaging as a means to predict disease progression, assess therapeutic responses, and gauge therapeutic outcomes.

### Tetraspanins and other membrane proteins that are sorted in exosomes

Exosomes are highly enriched in tetraspanin family members, including CD81, CD63, CD9, CD82, and CD37^39^. While these tetraspanins lack intrinsic enzyme-linked receptors or catalytic activity, they play crucial roles in facilitating the sorting of protein cargoes, particularly tetraspanin-interacting proteins, such as integrins^44^, ICAM-1^45^, IGFS-8^46^, major histocompatibility complex class II proteins^47^, and syndecan^22^.

The surfaces of exosomes harbor various other transmembrane proteins with scaffolding functions, including flotillin 1 and 2^48^, IL-6R^49^, EGFR, T-cell receptor^50^, chimeric antigen receptor^51^, GPCR receptors^52^, PD-L1^53^, TGFB^54^, and ADAM proteases^55^. In addition to transmembrane proteins, the surface of exosomes is also rich in many membrane-interacting proteins, especially proteins with glycosylphosphatidylinositol (GIP) anchors, such as proteoglycans, glypican-1^56^, DAF, and MAC-IP, which further augment the protein landscape of the exosome surface.

A variety of proteins, including small GTPases that contribute to exosome biogenesis and are attached via posttranslational prenylation, have been found on the inner leaflet of exosome membranes. Additionally, proteins myristoylated at the N-terminus, such as BASP-1 and Src signaling kinases, are among those that undergo sorting into exosomes. Similarly, N-terminal myristoylation is used for loading lentiviral gag into viruses or exosomes. Other posttranslational modifications, such as ubiquitination, SUMOylation, and phosphorylation, have also been implicated in cargo sorting^57^.

The abundant proteins within exosomes include molecular chaperones that interact with or are part of ESCRT complexes, including ALIX, TSG101, and syntenin. In addition to biogenesis-related proteins, exosomes are enriched in molecular chaperones such as HSP70, HSP90, and HSP20.

### Exosomes as RNA carriers

Despite the well-established process of cargo selection for transmembrane receptors through ubiquitylation and recognition by ESCRT components in exosome biogenesis, the mechanisms that govern the incorporation of cytoplasmic cargoes, including RNAs and RNA-binding proteins (RBPs), into exosomes have not been fully elucidated. Multiple reports using next-generation sequencing and microarray technologies to characterize RNA content in exosomes sourced from cell cultures, tissues, or biological fluids have revealed the enrichment of specific RNAs. These included mRNA fragments (≤1 kb in length), miRNAs, snRNAs, tRNA fragments, snoRNAs, mitochondrial RNAs (mtRNAs), piRNAs, vault RNAs (vtRNAs), and Y RNAs. Circular RNAs (circRNAs), rRNA fragments, and long non-coding RNAs (lncRNAs) have also been identified in exosomes^58,59^.

For the protein cargo, the current literature indicates that the RNA composition of exosomes varies depending on the cellular context. Emerging evidence suggests that RBPs play a pivotal role in orchestrating the selective sorting of various small RNAs, including miRNAs, tRNAs, Y RNAs, vault RNAs, and others, into exosomes. RBPs are primarily localized to sites of exosome biogenesis and function as adaptors between RNA cargo and exosome biogenesis machinery^14,60^ (Table 1). Generally, sorting mechanisms are classified as active RNA-loading processes^61^. In these processes, specific regulatory mechanisms are involved in actively selecting and incorporating certain RNAs into exosomes. Passive loading, on the other hand, is primarily driven by the intracellular concentration of a specific RNA, and the presence of a specific RNA in exosomes is largely dependent on the abundance of that RNA within the source cell^62^.

A distinctive category of active RNA loading is selective loading, which involves the specific sorting of particular RNA species in exosomes. This selective sorting has been demonstrated for miRNAs that possess specific motifs containing EXOmotifs, with CGGGAG being the strongest example. Conversely, miRNAs with CELL motifs, such as AGAAC, CAGU, or AUUA, tend to be retained within source cells^63,64^. Moreover, specific motifs have been characterized for several RBPs, including hnRNPA2B1 (GGAG, AGG, or UAG), hnRNPK (AsQGnA), SYNCRPI (GGCU), and FMRP (AAUGC). These motifs contribute to the selective incorporation of associated RNAs into exosomes, often regardless of the overall RNA composition of the source cells^65,66^. Additionally, 40 miRNA seed sequences were identified as motifs enriched in lncRNAs associated with prostate cancer exosomes. The selection of these miRNAs is governed by cell type-specific preferences, where specific GC-rich miRNA sequence elements are recognized by RBPs.

Given the presence of more than 4,000 identified RBPs in the human genome at present^67^ (a list of RBPs is available in the online database RBPbase v0.2.0 Alpha, https://rbpbase.shiny.embl.de) and given that RBPs constitute 25% of the EV protein content, RBPs are undoubtedly key players in the active sorting of RNA into EVs. Among these RBPs, hnRNPA2B1 has been extensively reported to be a pivotal factor in regulating the loading of numerous miRNAs^68,69^ (Table 1). Although the exact mechanism through which hnRNPA2B1 engages with the exosome biogenesis machinery has not been determined, its association with intracellular ceramide-rich MVB structures suggests a potential link to the nSMase2-ceramide pathway in exosome biogenesis. Intriguingly, the role of hnRNPA2B1 in facilitating colorectal cancer liver metastasis and bladder cancer lymphatic metastasis is supported by its regulation of exosomal sorting of tumor cell miRNAs and lncRNAs such as AFAP1-AS1, AGAP2-AS1, H19, and LNMAT2, respectively^70,71^ (Table 1). Notably, studies involving RBP YBX1 have indicated its potential role in the sorting of a variety of small RNAs (miRNAs, tRNAs, Y RNAs, and vault RNAs) into exosomes, as demonstrated by the coprecipitation of YBX1 with RNAs and the subsequent reduction in the levels of these associated RNAs following YBX1 deletion^72^. Similarly, the RBP La directly binds miRNAs and preferentially directs them into high-density vesicles while excluding low-density counterparts.

RNA sorting into exosomes extends beyond the binding of specific RNA sequence motifs and encompasses various RNA or RBP modifications. For example, SUMOylation has been implicated in the loading of miRNAs into exosomes, with hnRNPAB1 being the key mediator of this process^70^. Phosphorylation has also been linked to exosome cargo packaging, as exemplified by exosomal 5’pppRNA during latent EBV infection^73^. Additionally, LC3 conjugation has been associated with the loading of small noncoding RNA species, including snoRNAs and miRNAs, within the framework of the secretory autophagy pathway. Similarly, LC3 conjugation has been described for hnRNPK- and SAFB-mediated loading of these small ncRNA species.

Although the importance of RBPs in mediating RNA sorting into exosomes is evident, the specific mechanisms by which these RBPs interact with the exosome biogenesis machinery are unclear. Notably, FMRP is recruited to MVBs through interactions with the RAB-interacting lysosomal protein and HRS. This intriguing connection links FMRP-mediated miRNA sorting with the ESCRT-mediated process of exosome biogenesis^74^.

Moreover, membrane contact sites between the ER and MVBs have emerged as crucial locations for the generation of RNA-enriched exosomes. A recent study identified integral membrane proteins, namely, the ER tether protein VAP-A and the ceramide transport protein CERT, as crucial components in the biogenesis of both large and small EVs loaded with RNA and RBPs^75^. Given the role of the ER as a structural scaffold for various RNA granules, the regulation of RBP-RNA complexes localized to the ER could play a significant role in driving the formation of RNA-containing exosomes. These findings illuminate the multifaceted landscape of RNA sorting into exosomes and highlight the intricate interplay between RBPs, RNA modifications, and the exosome biogenesis machinery in shaping the cargo profile of these versatile vesicles.

### Exosomes as vehicles for DNA and mtDNA transport

While RNA has been extensively investigated as a cargo molecule in exosomes, considerably less research has been dedicated to understanding the origin and clinical significance of DNA in these vesicles. Various DNA species, including genomic dsDNA^76^, dsDNA-binding histone proteins^77^, ssDNA, mitochondrial DNA (mtRNA)^78^, and viral DNA, are associated with exosomes. The presence of DNA within exosomes has generated significant interest, particularly in the context of liquid biopsy biomarker analysis^79,80^. Interestingly, recent studies employing advanced imaging and biochemical analysis have revealed the presence of DNA both on the surface and within exosomes. However, the mechanisms orchestrating the integration of DNA into exosomes have not been fully elucidated.

Traditionally, the majority of EV-containing DNA was thought to originate from apoptotic bodies. However, ongoing investigations have revealed a more intricate scenario in which nonapoptotic EVs have emerged as carriers of DNA. Notably, a substantial portion of DNA is found within large EVs, including both apoptotic and nonapoptotic subtypes. This phenomenon is evident in studies comparing large EVs and exosomes from prostate cancer cells and patient plasma^80^. Intriguingly, large nonapoptotic EVs seem to accumulate more DNA, especially in cancer cells with nuclear shape instability, suggesting that these cells play a role in encapsulating and exporting cytosolic DNA that is generated due to genomic instability in rapidly dividing cancer cells.

Furthermore, recent research has suggested that DNA secretion via exosomes exerts a crucial cytoprotective function by alleviating cellular stress associated with the accumulation of deleterious cytoplasmic DNA and micronuclei. Consequently, therapy-induced DNA damage in cancer cells may contribute to exosome-mediated DNA packaging. However, the mechanisms governing DNA recruitment into exosomes have not been elucidated. Understanding the process of DNA entry into MVBs remains a challenge. Nevertheless, some reports have successfully demonstrated the presence of DNA within exosomes^16^. Importantly, senescence and DNA damage have been linked to increased DNA levels in MVBs and exosomes, thus offering a potential explanation for the prevalence of DNA in exosomes, particularly in the context of exosomes derived from cancer cells.

This evolving field holds great promise for shedding light on the role of exosomes as carriers of DNA, with potential implications for diagnostic and therapeutic applications. However, further research is needed to unravel the intricate mechanisms underlying DNA sorting into exosomes and to fully understand the clinical relevance of DNA cargo within these versatile vesicles.

### Exosomes as lipid carriers

Lipids constitute another crucial class of macromolecules that are packaged into exosomes. These vesicles are particularly rich in various lipids, including cholesterol, phosphatidylcholine, phosphatidylserine, sphingomyelin, and ceramide. These lipids play diverse and essential roles in exosome biogenesis, uptake, and the functional impact of exosomes on recipient cells.

In the context of cancer, in an extensive, large-scale lipidomic analysis, researchers quantified >200 lipid species in exosomes derived from PC3 prostate cancer cells. This analysis revealed that certain lipids, including cholesterol, sphingomyelin, and glycosphingolipids such as ceramides and phosphatidylserine, were more abundant in exosomes than in other phospholipids and were generally less abundant in exosomes^81^. Furthermore, exosomes isolated from the urine of prostate cancer patients exhibit higher levels of specific lipids, notably ceramides, than urine-derived exosomes from healthy patients. This observation suggests the potential utility of these lipids as fluid-based biomarkers^82^. However, a separate study reported reduced ceramide levels in urine exosomes from stage 2 benign prostate hyperplasia patients compared to urine exosomes from stage 3 prostate cancer patients, thus complicating the potential use of ceramides as biomarkers. Additionally, exosomes from colorectal cancer, glioblastoma, and hepatocellular carcinoma cells also demonstrated cholesterol, sphingomyelin, and phosphatidylserine enrichment in contrast to their parent cells^83,84^. These findings highlight commonalities in the lipid content of exosomes across different cancer types. Further research aimed at elucidating the mechanisms underlying lipid packaging in exosomes and uncovering the functional roles of these lipid components holds great promise for enhancing the potential of these components as biomarkers and therapeutic tools.

## Therapeutic insights into small-molecule compounds that target exosome biogenesis and secretion in cancer

Exosomes facilitate fundamental cell-to-cell communication, a process that is conserved across all life forms. These minuscule vesicles play pivotal roles in a wide range of (patho-)physiological processes, including maintaining cellular balance, transmitting infections, driving cancer progression, and contributing to cardiovascular diseases. Consequently, strategies aimed at blocking the release or biogenesis of exosomes or regulating exosomal cargo hold promise for slowing the progression of diseases, particularly cancers^85,86^.

The role of exosomes in systemic aspects of human disease, particularly cancer, has garnered significant attention. Exosomes are present in all body fluids, and their cargo signature can be used to predict cancer type, stage, and therapeutic response^43,87^. However, the importance of tumor-derived exosomes in shaping the tumor microenvironment homeostasis should not be underestimated. Experimental studies have consistently shown that inhibiting exosome production in cancer and stromal cells reduces cancer growth and metastasis^88^. This finding underscores the critical role of exosome secretion in cancer development.

Exosomes influence all aspects of cancer progression, including carcinogenesis, host-microbiota interactions, metastasis, immune responses, drug resistance, and the formation of premetastatic niches in distant organs^89^. Various factors, such as cellular stress (e.g., hypoxia, nutrient deprivation, and oxidative stress) and the presence of oncogenes, such as Src, EGFRvIII, and KRAS, can impact the composition of exosomal cargo^25,90,91^. For example, the presence of Src in cells increases the release of exosomes containing proinflammatory proteins, while the presence of KRAS leads to an increase in the number of exosomes containing proteins and miRNAs involved in cell growth and survival. Modulating signaling in exosome biogenesis and secretion pathways can affect exosome contents, suggesting that regulating exosomal composition is a promising avenue for developing new cancer therapies.

However, research into the intricate mechanisms governing the sorting of exosomal cargo is in its nascent stages, and specific inhibitors have not yet been identified. The formation of exosomes is also intricately intertwined in this process. Here, we introduce the various small molecules known to be involved in exosome formation, including 33 inhibitors that were assessed based on the pathways of exosome biogenesis or secretion that they primarily target. These inhibitors play a crucial role in regulating exosome secretion and have shown promise in the development of new cancer therapies. Table 2 summarizes these pathways and their corresponding inhibitors.

**Table 2:** Potential exosome inhibitors and their targets for cancer treatment.

Inhibitor	Targets	Pathways	Cancer cell types	Refs.
Exosome biogenesis				
SyntOFF	Syntenin	Syndecan-Syntenin-Alix	Breast cancer	^98^
Imipramine	Acid sphingomyelinase (aSMase)	aSMase	Prostate cancer	^93^
GW4869	Neutral sphingomyelinase (nSMase)	nSMase-ceramide pathway	Breast cancer, prostate cancer, melanoma, glioma, and myeloma	^92,95^
Spiroepoxide	Neutral sphingomyelinase (nSMase)	nSMase-ceramide pathway	-	^92^
Simvastatin	HMG-CoA reductase	Cholesterol synthesis	-	^94^
Glibenclamide(Glyburide)	ATP-binding cassette transporter	Potassium channels block	Breast cancer, prostate cancer	^93^
Indomethacin	Cyclooxygenase I/II	ABCA3 and lipid transporter	-	^95^
Exosome release				
Y27632	Rho-associated protein kinases (ROCK)	Cytoskeletal reorganization	Breast cancer	^95^
U0126	MEK1/2	Ras/Raf/ERK signaling	Prostate cancer	^95^
Manumycin A	Farnesyltransferase (FTase)	Ras/Raf/ERK1/2 signaling	Prostate cancer	^97^
Tipifarnib	Farnesyltransferase (FTase) and Rab	Ras/Raf/ERK1/2 signaling	Rhabdomyosarcoma, prostate cancer	^97^
CPPG	GRM3	RAB27a and Alix	Multiple myeloma	^99^
LY341495	GRM3	RAB27a	Breast cancer	^99^
Sulfisoxazole	Endothelin receptor A	Multiple process	Breast cancer	^95,100^
Macitentan	Endothelin receptor A	Multiple process	Breast, colon and lung cancer	^101^

- : no tested in cancer cells

Among these inhibitors are those that target sphingomyelinases, such as imipramine, GW4869, cambinol, and spiroepoxide. The latter three selectively inhibit neutral sphingomyelinase (nSMase or aSMase). Each of these inhibitors operates through distinct mechanisms, ultimately reducing exosome secretion by blocking ceramide-modulated inward budding of MVBs and the subsequent release of exosomes from MVBs^92^. Additionally, inhibitors such as glyburide (glibenclamide) target ATP-sensitive K+ channels, while others, such as indomethacin, target ATP-binding cassette transporters. These inhibitors regulate cellular cholesterol and phospholipid concentrations, ultimately inhibiting the release of MVs and exosomes^93^. Simvastatin, an HMG-CoA reductase inhibitor, prevents the synthesis of cholesterol and decreases the intracellular concentrations of exosome-associated proteins such as ALIX, CD63, and CD81^94^. Furthermore, proteins involved in cytoskeletal organization are key targets for various inhibitors, as they are essential for both exosome release and endocytic processes. Y27632, a competitive inhibitor of the ROCK family, which includes ROCK1 and ROCK2, plays a crucial role in this context. Y27632 competes with ATP for binding to the catalytic sites of ROCK1 and ROCK2, resulting in a decrease in MV and exosome-sized secretion. Additionally, inhibitors targeting protein kinases, such as Y27632, U0126, imatinib, and dasatinib, are effective at preventing the activation of ERK, which is necessary for microvesiculation^95^.

Several inhibitors target the ESCRT-dependent pathway of exosome production. These inhibitors act on Ras farnesyltransferase enzymes by inhibiting their activation and disrupting the Ras/Raf/ERK1/2 signaling pathway, which is crucial in the ESCRT-dependent pathway related to exosome biogenesis^96,97^. SyntOFF binds to the PDZ domain of syntenin, thereby disrupting the syndecan-syntenin-ALIX pathway^98^. This disruption results in reduced proliferation and metastasis in breast cancer patients and decreased exosome secretion. In multiple myeloma and bone marrow stromal cells, the glutamate antiporter system facilitates glutamate export and promotes exosome secretion by upregulating the expression of Rab27a, TSG101, ALIX, and VAMP7. This mechanism contributes to bortezomib resistance in multiple myeloma. Targeting GRM3 with the antagonist (RS) alpha-cyclopropyl-4-phosphonophenylglycine (CPPG) reduces exosome secretion and overcomes drug resistance in multiple myeloma^99^. Recently, endothelin A receptor (ETA) antagonists, such as sulfisoxazole (SFX) and macitentan (MAC), have emerged as new agents for inhibiting exosome secretion. SFX, which was originally an antibacterial drug, has been shown to inhibit components within or related to the ESCRT-dependent pathway, including ALIX and VPS4B, as well as some RAB proteins in breast cancer cell lines^95,100^. Another ETA antagonist, MAC, inhibits exosomal PD-L1 secretion^101^. This inhibition reduces the number of exosomes that bind to PD-1 on T cells, thereby enhancing antitumor immunity by revitalizing exhausted T cells. These findings suggest that targeting various steps of exosome biogenesis and secretion holds promise for the development of effective drugs.^99,102^

## Exosome engineering for selective cargo sorting: active vs. passive processes

**Fig. 3:**
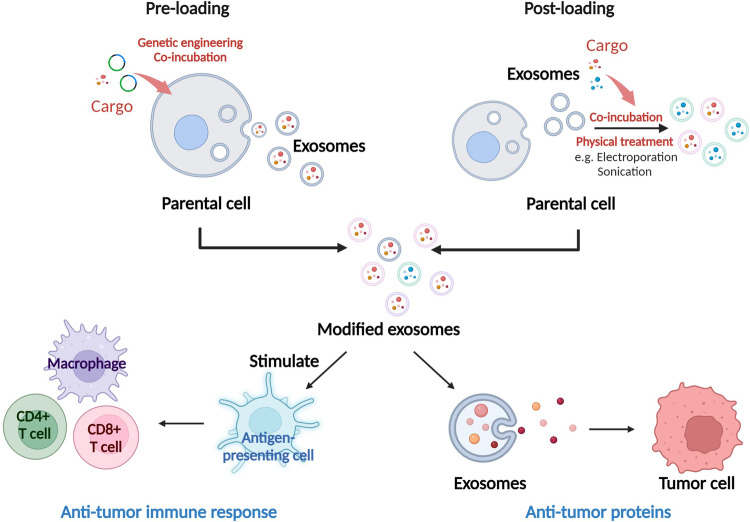
Exosome engineering strategies for loading cancer therapeutic cargo into exosomes. Exosomes can be engineered to target internal and modified surface cargoes for cancer therapy. Cargo loading strategies are achieved by incubating therapeutic agents (e.g., pharmacological inhibitors, miRNAs, and recombinant proteins) directly with isolated exosomes (post-loading) or by exposing them to exosome-secreting donor cells, followed by the isolation of loaded exosomes (pre-loading). Post-loading methods require physical treatments to disrupt membrane integrity and allow the cargo to enter the interior of exosomes. Alternatively, exosome-producing cells with genetic expression constructs that encode therapeutic cargoes linked to an exosome sorting domain can be generated. This leads to sorting therapeutic cargo into exosomes. Modified exosomes containing tumor antigens can stimulate antigen-presenting cells and drive antitumor immune responses in the human body. Engineered exosomes can also directly release antitumor cargo to attack cancer cells.

Exosomes are reservoirs of diverse biomolecular cargo, including lipids, proteins, and nucleic acids. Recent advancements in genetic tools, such as RNAi, CRISPR-Cas9, and recombinant DNA technology, coupled with enhanced exosome characterization techniques, have begun to unveil the intricate mechanisms underlying cargo sorting into exosomes (Fig. 2). Currently, research on exosome modification predominantly revolves around two methods: active and passive approaches^103^. Active strategies involve the integration of target substances during exosomal biogenesis, often through genetic cell modifications. Passive approaches involve the loading of a combination of exogenous substances after exosome secretion via techniques such as electroporation or chemical conjugation (Fig. 3). Key objectives in exosome bioengineering include accurate cargo loading and enhancing exosomal targeting.

The primary components of exosomal cargo typically include small RNAs and proteins. Exosomal cargo ‘selection’ or ‘sorting’ refers to processes that locally enrich cargo molecules during nascent exosome formation. Exosome selection and sorting mechanisms involve exosome biogenesis machinery that is responsible for capturing and aggregating cargoes. In active loading, parental cells undergo genetic engineering to overexpress a desired protein fused with an exosome scaffold, which is subsequently integrated into secreted vesicles during biogenesis^104^. Despite extensive exploration of endogenous scaffolds for exosomal cargo engineering, achieving versatile strategies for loading biotherapeutic cargo into exosomes remains a challenge due to the diversity of proteins that participate in exosome biogenesis and cargo loading. Importantly, the choice of cargo fusion partner profoundly impacts loading efficiency. It is essential to consider that fusion of a specific cargo with one exosome biogenesis regulator may result in suboptimal loading into exosomes, whereas fusion with another may yield superior engineering performance. Ideally, exosome engineering scaffolds should enable the efficient packaging of multiple copies of proteins without disrupting the exosome biogenesis pathway or the luminal content of exosomes. Therefore, various studies have systematically compared different exosome-sorting domains for endogenous engineering using the latest innovations in exosome characterization to determine the efficiency of these methods at the single-exosome level. In a recent study, 150 enhanced green fluorescent protein molecules were loaded per vesicle using TSPAN14 as the exosome scaffold^105^. Other identified exosome-sorting domains include the syntenin^106^, ARRDC1, BASP-1, syndecan-1, and HIV-I Nef proteins. To facilitate the post-encapsulation release of biotherapeutic cargo, advanced systems, such as light-induced dimerization systems^107^ and small-molecule-controlled protein associations^108^, have been developed. By leveraging these innovations in endogenous exosome engineering, exosomes have been bioengineered for delivering CRISPR/Cas9^109^, the IkBa superrepressor^110^, Cre recombinase^111^, and lysosomal enzymes (Fig. 3).

Following the discovery of functional RNA transfer via exosomes^112^, substantial efforts have been directed toward creating bioengineered exosomes as nanosized biomimetic delivery agents for therapeutic nucleic acid cargo. Encapsulating RNA into exosomes has been approached through both brute-force overexpression and engineered targeting. Overexpressing Cre recombinase mRNA within donor cells results in its presence within exosomes, albeit generally with low efficiency. Notably, recent progress has been made with siRNA sequences incorporated into a Dicer-independent pre-miRNA stem–loop (premiR-451), which has demonstrated enhanced loading efficiency using miRNA sorting machinery^113^. For exosome loading of nucleic acid cargo, strategies similar to those discussed earlier for therapeutic proteins involving the fusion of a nucleic acid-binding protein with an exosome-sorting protein have been adopted. This methodology has successfully facilitated the loading of small RNAs^114^ as well as longer RNA species such as mRNAs^115^. Consequently, in experimental scenarios, careful management of co-delivered passively loaded proteins is crucial. In therapeutic applications, co-delivering mRNA, along with therapeutic proteins, can confer advantages. A recent approach elegantly combined endogenous and exogenous exosome loading strategies. The approach involved expressing a DNA aptamer within producer cells to sequester target mRNAs while efficiently loading this complex into exosomes via a CD9-zinc finger fusion protein. To release the mRNA from the bound DNA aptamer, purified mRNA/DNA aptamer-loaded exosomes were electroporated to encapsulate a Klenow fragment exonuclease. This modification enabled mRNA translation in recipient cells both in vitro and in vivo^116^.

## Exosomes as promising delivery systems for anticancer drugs and future perspectives

Exosomes are natural vesicles secreted by various cells in the body and serve as essential carriers of information between cells. Exosomes are innate carriers of functional genetic information that exhibit low toxicity and widespread tissue distribution in vivo. They also possess more than 30 times greater cellular uptake than alternative delivery systems such as nanoparticles or liposomes^117^. Additionally, exosomes exhibit resistance to harsh environmental conditions, such as low blood pH, making them promising biocarriers for drugs, nucleic acids, and imaging agents in cancer therapy. The potential of exosomes as delivery systems for anticancer drugs, particularly compounds with low solubility and limited off-target delivery, is of significant interest.

Manipulating exosomes by inhibiting their biogenesis or modifying their surface membranes and loading them with cargo, such as proteins, nucleic acids, or drugs, has demonstrated remarkable potential in reducing tumor progression and metastasis. Notably, exosomes are at the forefront of clinical trials for diverse therapeutic applications; there are 118 ongoing trials as of 2022 (https://www.clinicaltrials.gov/). While preclinical studies have shown promising therapeutic efficacy, ongoing clinical trials are further evaluating the potential of these agents. Table 3 provides a comprehensive list of clinical trials utilizing exosomes as therapeutic tools. However, despite the immense promise of EVs for therapeutic and diagnostic purposes, several significant challenges must be addressed.

**Table 3:** Overview of clinical trials using exosomes for cancer treatment.

Sr. no.	Disease	Exosome source	Status	Remarks	Clinical trial identification	Ref.
1	Pancreatic cancer	Ultrasound-guided portal venous blood exosome	Recruiting	Safety of sampling portal venous blood, analyzing mRNA markers	NCT03821909	^118^
2	Colon cancer	Curcumin conjugated with plant exosomes	Active, Phase I	Comparing exosome-loaded curcumin on immune modulation, phospholipid profile of normal and malignant patients	NCT01294072	Not available
3	Sarcoma	Blood samples	Recruiting	Evaluation of cancer pathogenesis, progression, and treatment efficacy of exosomes	NCT03800121	^119^
4	Prostate cancer	Urine exosomes	Completed	Validation of non-digital rectal examination (DRE) exosome gene expression test of prostate cancer in biopsy	NCT02702856	^120^
5	Pancreatic cancer	Blood samples from patients	Active, not recruiting	Exosome purification for RNA sequencing and proteomics	NCT02393703	^121^
6	Lung metastasis osteosarcoma	Blood samples	Recruiting	Identification of levels of circulating exosomal RNA with or without lung metastasis	NCT03108677	^122^
7	Gallbladder carcinoma	Exosomal blood samples	Recruiting	Establishing a correlation between exosome biomarkers and gallbladder carcinoma	NCT03581435	Not available
8	Stage IV pancreatic adenocarcinoma	Mesenchymal stromal cells-derived exosomes with KRAS G12D siRNA	Phase-I, not recruiting	Mesenchymal-derived exosomes with KRAS G12D in treating individuals with pancreatic cancer with KRAS G12D mutation	NCT03608631	^5^
9	Pancreatic ductal adenocarcinoma	Portal vein blood	Completed	Test 3 CTC isolation methods and analyses for onco exosomes in pancreatic cell culture media by flow cytometry	NCT03032913	^123^
10	Oral mucositis, head and neck cancer	Grape extract exosomes	Active, phase-I, not recruiting	The ability of plant exosomes to prevent oral mucositis in head and neck cancer	NCT01668849	Not available
11	Non-small cell lung cancer(NSCLC)	Plasma exosomes	Not recruiting	New radiotherapy combined with immunotherapy	NCT02890849	^53^
12	Non-small cell lung cancer(NSCLC)	Dendritic-derived exosomes	Completed Phase-2	No induction of T cells monitored in patients	NCT01159288	Not available
13	Thyroid cancer	Urine exosomal thyroglobulin and galectin3	Active, not recruiting	Identifying urinary exosomal proteins (thyroglobulin and galectin 3)	NCT03488134	^124^
14	Colon cancer	Blood samples	Recruiting	Novel ways of diagnosing and predicting the spread to other organs, such as liver	NCT03432806	Not available
15	Prostate cancer	Urine samples	Active, not recruiting	Validated urine test to predict the incidence of high-grade prostate cancer in initial prostate biopsy	NCT03235687	^121^
16	Triple-negative breast cancer	Serum exosomes	Phase-I, recruiting	Assessing response to pembrolizumab in the primary tumor, circulating lymphocytes	NCT02977468	Not available
17	Thyroid cancer	Urine exosomes	Active, not recruiting	Evaluation of new therapeutic mechanisms and medications for poorly differentiated or anaplastic thyroid cancer	NCT02862470	Not available
18	Lung cancer	Blood samples	Recruiting	Drug efficacy, surgical effect evaluation, recurrence monitoring, prognosis judgment, molecular differentiation by analyzing blood ctDNA	NCT03317080	^125^

## Technical challenges in exosome-based therapies

There are various technical obstacles in developing exosome-based therapies, including isolation techniques, characterization methods, and the standardization of clinically suitable exosome preparations. While conventional low-throughput techniques such as ultracentrifugation are widely used for exosome isolation, emerging techniques such as tangential flow filtration and size exclusion chromatography offer the potential to handle larger volumes of exosomes. Scaling up production for clinical applications involving bioreactors and media supplements, such as fetal bovine serum containing exosomes presents additional complexities and underscores the critical importance of rigorous characterization. Standardization is paramount, particularly in defining exosome function, determining effective doses, and unraveling exosome mechanisms of action.

Confronting the inherent heterogeneity of exosomes and refining isolation protocols to select vesicles with optimal functional activity is essential. The development of potency assays capable of gauging the effectiveness of specific exosome populations is a priority and will facilitate the regulatory approval process. Concerns regarding the loading of exogenous cargo into exosomes, which could interfere with endogenous cargo and give rise to off-target effects, especially in complex conditions such as cancer, have been noted. Identifying the safest cell source for exosome isolation to mitigate immunogenicity and unwanted cargo is critical and will require the use of appropriate preclinical models and meticulous cargo selection.

Efforts to target exosomes to specific sites for therapeutic intervention necessitate a deeper understanding of the delivery mechanisms. Balancing natural processing with payload targeting to minimize off-target effects is a formidable hurdle. While engineering exosomes to increase tropism for specific cells or organs has shown promise, the production of therapeutically relevant quantities of exosomes remains challenging. Recent advancements involving the incorporation of nanobody fragments or RNA aptamers on exosome surfaces have shown some degree of targeting specificity.

Moreover, exosome-based biomarkers, whether based on proteins, nucleic acids, lipids, or glycans, hold immense potential for addressing diseases such as cancer and neurological conditions. However, the translation of these discoveries into clinical applications requires technological innovations, particularly in the development of high-throughput exosome isolation methods and standardized assays. Cross-validation of exosome disease biomarkers across different laboratories and sample cohorts will expedite the transition from the laboratory to clinical practice.

## Concluding remarks

Gaining a deeper understanding of the intricate machinery governing cargo selection and packaging during exosome biogenesis holds great promise for advancing our comprehension of intercellular signaling within the human body. This research has the potential to shed light on fundamental questions, including why distinct cell types produce unique exosome profiles and how external environmental conditions and the cellular state influence exosome composition. Moreover, considering the involvement of exosomes in numerous disease contexts, these insights offer promise for the development of targeted therapeutic interventions that precisely diagnose and regulate disease-associated exosomes.

Furthermore, elucidating the cargo selection and packaging processes is pivotal for harnessing exosomes as a versatile platform for therapeutic intervention. Researchers can engineer exosomes to transport specific cargoes, such as therapeutic drugs or genetic material, and direct them to specific cells or tissues. This precision is paramount for achieving highly desirable therapeutic outcomes, as it minimizes off-target effects and enhances treatment efficacy. Continued investigations into exosome biology and engineering hold the potential to significantly impact health care and enhance our capacity to address various diseases.
